# What zinc supplementation does and does not achieve in diarrhea prevention: a systematic review and meta-analysis

**DOI:** 10.1186/1471-2334-11-122

**Published:** 2011-05-12

**Authors:** Archana B Patel, Manju Mamtani, Neetu Badhoniya, Hemant Kulkarni

**Affiliations:** 1 Lata Medical Research Foundation, Nagpur, India; 2 Indira Gandhi Government Medical College, Nagpur, India; 3 The University of Texas Health Science Center at San Antonio, Texas, USA

## Abstract

**Background:**

Prevention of diarrhea has presented indomitable challenges. A preventive strategy that has received significant interest is zinc supplementation. Existing literature including quantitative meta-analyses and systematic reviews tend to show that zinc supplementation is beneficial however evidence to the contrary is augmenting. We therefore conducted an updated and comprehensive meta-analytical synthesis of the existing literature on the effect of zinc supplementation in prevention of diarrhea.

**Methods:**

EMBASE^®^, MEDLINE ^® ^and CINAHL^® ^databases were searched for published reviews and meta-analyses on the use of zinc supplementation for the prevention childhood diarrhea. Additional RCTs published following the meta-analyses were also sought. Effect of zinc supplementation on the following five outcomes was studied: incidence of diarrhea, prevalence of diarrhea, incidence of persistent diarrhea, incidence of dysentery and incidence of mortality. The published RCTs were combined using random-effects meta-analyses, subgroup meta-analyses, meta-regression, cumulative meta-analyses and restricted meta-analyses to quantify and characterize the role of zinc supplementation with the afore stated outcomes.

**Results:**

We found that zinc supplementation has a modest beneficial association (9% reduction) with incidence of diarrhea, a stronger beneficial association (19% reduction) with prevalence of diarrhea and occurrence of multiple diarrheal episodes (28% reduction) but there was significant unexplained heterogeneity across the studies for these associations. Age, continent of study origin, zinc salt and risk of bias contributed significantly to between studies heterogeneity. Zinc supplementation did not show statistically significant benefit in reducing the incidence of persistent diarrhea, dysentery or mortality. In most instances, the 95% prediction intervals for summary relative risk estimates straddled unity.

**Conclusions:**

Demonstrable benefit of preventive zinc supplementation was observed against two of the five diarrhea-related outcomes but the prediction intervals straddled unity. Thus the evidence for a preventive benefit of zinc against diarrhea is inconclusive. Continued efforts are needed to better understand the sources of heterogeneity. The outcomes of zinc supplementation may be improved by identifying subgroups that need zinc supplementation.

## Background

Preventing childhood diarrheas is difficult but important, especially in developing countries. An intensely studied and evaluated effort in this direction focuses on zinc supplementation since this micronutrient is believed to play a critical role in the pathogenesis of childhood diarrheas. Zinc is one of several important trace elements that have far-reaching effects on multiple organs and systems and serves over 300 biological functions [1]. Therefore it is argued that chronic zinc deficiency may increase diarrhea susceptibility.

On the basis of the substantial body of biological evidence, it can be envisaged that zinc supplementation to children with zinc deficiency may help arrest or at least lessen diarrheal incidence and prevalence. For example, owing to the differential distribution of the prevalence of zinc deficiency, the World Health Organization and WHO/UNICEF recommends therapeutic zinc supplementation for diarrhea only in developing countries but not in developed countries [2,3]. While guidelines for zinc supplementation during an episode of diarrhea are clearer, there is currently no unified view about the need to provide zinc supplementation as a preventive measure to curb the incidence and prevalence of diarrhea. Three published meta-analyses have thus far evaluated the use of zinc supplementation in the scenario of diarrhea prevention[4-6]. The first meta-analysis by Bhutta et al[5] included seven studies and showed an overall 18% reduction in incidence of diarrhea attributable to zinc supplementation. The second meta-analysis included 15 studies[4], reported a slightly lesser but still significant benefit of a 14% reduction in incidence of diarrhea. The most recent meta-analysis[6] included 24 studies with 33 distinct comparisons. It showed a 20% reduction in incidence of diarrhea. Interestingly, the meta-analyses by Aggarwal et al[4] reported a statistically significant heterogeneity across the published studies.

In spite of these elegant reports, there exist several gaps in the current understanding of the potential benefit of zinc supplementation as a prophylactic measure. First, the observed heterogeneity across published studies somewhat questions the reliability of the summary effect measures that have been heretofore ascribed to zinc supplementation. Second, several additional trials [7-12] have been published since the last meta-analysis and a formal synthesis inclusive of the newer evidence is currently lacking. Third, the published meta-analyses have reported the influence of zinc supplementation on overlapping as well as different diarrheal outcomes but a single compiled report on these different outcomes is not available. Finally, the temporal relevance of zinc supplementation as a public health measure against childhood diarrhea is not known. We therefore conducted the present study with the following three aims: i) to update the meta-analysis of preventive use of zinc in children for most commonly reported diarrheal outcomes; ii) to understand the sources of heterogeneity, if any; and iii) to understand if the evidenced-based benefit attributable to zinc has changed over time.

## Methods

### Data Extraction

**Figure 1:**
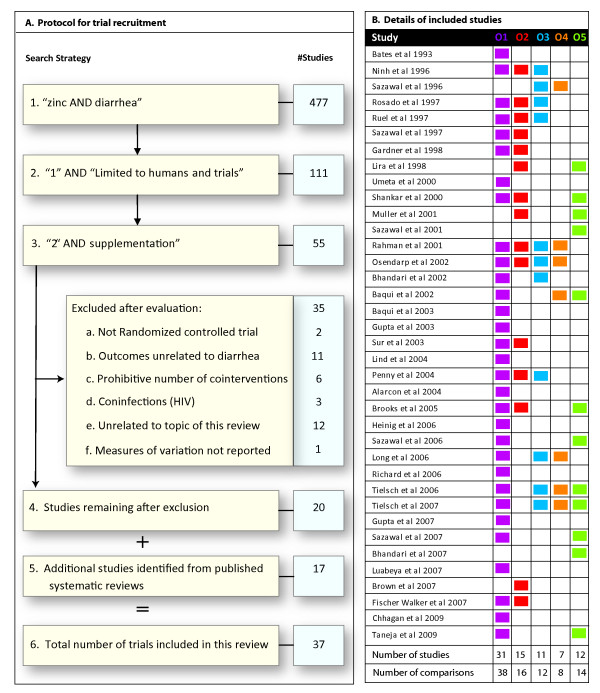
**Studies included in this review**. **(A) **Flowchart showing the search strategies and the exclusion criteria used to locate the relevant studies in this review. **(B) **This panel lists the studies included in this synthesis and the outcomes (O1-O5) reported by each study. The outcomes are color-coded and the colors are consistently used in the rest of the paper. A colored square in the grid indicates that the specified outcome was reported by a trial while a blank square in the grid indicates that the outcome was not reported. The outcomes are as follows: O1, incidence of diarrhea; O2, prevalence of diarrhea; O3, incidence of persistent diarrhea; O4, incidence of dysentery; O5, mortality. Total number of studies and comparisons (when more than one zinc regimen was compared to the same reference group) are indicated at the bottom for each outcome.

We attempted to include all the recent published randomized controlled trials (RCT) that have been published after the three meta-analyses[4-6] were published. We also aimed to include additional past RCTs which may not been included in the previous meta-analyses. The search strategy and the search protocol are detailed in Figure 1A. To identify these studies we again searched the EMBASE^®^, CINAHL^® ^and MEDLINE^® ^databases for recently published trials on zinc supplementation. We searched using the following keywords: "zinc" and "diarrhea" and "supplement" limited to "humans" and "trials". We included studies that gave zinc supplementation for at least two weeks, had a length of follow-up of at least four weeks and in which the relative risk estimates were either reported or computable. All trials that provided zinc supplements with or without other nutrients versus the same preparation minus zinc were included. Trials that began as therapeutic trials to treat acute or persistent diarrhea and followed the children for prevalence or incidence of subsequent diarrhea were also included, provided they fulfilled the inclusion criteria stated above. As shown in Figure 1A, we excluded trials that did not conform to a randomized controlled trial design; reported outcomes unrelated to diarrhea; had prohibitive number of co-interventions or co-infections or were not directly related with the topic of this review. Our exclusion criteria resulted in the exclusion of four trials that were included in previous meta-analysis for following reasons: human immune-deficiency co-infection [13], relative risk not computable [14], outcome unrelated to diarrhea [15] and several co-interventions [16].

Our search strategy identified a total of 37 trials [7-12,17-47], the details of which are listed in Table 1. After locating the studies, at least three investigators independently evaluated each trial and inputs from these sources were collated into the final data set. All the authors independently reviewed the studies; any discrepancies in the study evaluations were discussed and resolved during one-to-one meetings. The report of this review is provided in line with the PRISMA^® ^guidelines (http://www.prisma-statement.org/index.htm) - the checklist and flowchart are provided separately (see Additional Files 1 and 2).

**Table 1:** Baseline characteristics of the studies included in the systemic review

#	Study	Country	Age (m)	Sample Size†		Zinc Supplementation				Plasma Zinc (μg/dl)	
						
				Zn	No Zn	Dose (mg)	Frequency/wk	Duration (wk)	Salt	Zn	No Zn
1	Bates et al 1993	The Gambia	7-28	55	54	70	2	60	*	-	-

2	Ninh et al 1996	Vietnam	4-36	73	73	10	1	22	Sulphate	-	-

3	Sazawal et al 1996	India	6-35	286	293	10	7	26	Gluconate	64.7	65.0

4	Rosado et al 1997	Mexico	18-36	54	55	20	7	54	Methionate	-	-

5	Ruel et al 1997	Guatemala	6-9	45	44	10	7	28	Sulphate	97.4	95.8

6	Sazawal et al 1997	India	6-35	298	311	10	7	26	Gluconate	-	-

7	Gardner et al 1998	Jamaica	6-24	31	30	5	7	12	Sulphate	64.6	65.0

8	Lira et al 1998	Brazil	0-6	139	66	5	7	8	Sulphate	-	-

9	Umeta et al 2000	Ethiopia	6-12	100	100	10	7	26	Sulphate	78.0	70.3

10	Shankar et al 2000	Papua New Guinea	6-60	136	138	10	7	46	Gluconate	70.4	70.2

11	Muller et al 2001	Burkina Faso	6-31	356	353	12.5	7	26	Sulphate	-	-

12	Sazawal et al 2001	India	0	581	573	5	7	35	Sulphate	-	-

13	Rahman et al 2001	Bangladesh	12-35	325	161	20	7	2	-	-	-

14	Osendarp et al 2002	Bangladesh	1	152	149	5	7	24	Acetate	66.9	75.9

15	Bhandari et al 2002	India	6-30	1241	1241	10	7	16	Gluconate	-	-

16	Baqui et al 2002	Bangladesh	3-59	3974	4096	20	7	2	Acetate	-	-

17	Baqui et al 2003	Bangladesh	6	323	157	20	1	26	Acetate	-	-

18	Gupta et al 2003	India	6-41	186	94	10	7	16	Sulphate	-	-

19	Sur et al 2003	India	0-12	50	50	5	7	52	Sulphate	-	-

20	Lind et al 2004	Indonesia	6-12	340	170	10	7	26	Sulphate	-	-

21	Penny et al 2004	Peru	6-36	276	136	20	7	26	Gluconate	-	-

22	Alarcon et al 2004	Peru	6-35	111	224	0.7	6	18	Sulphate	-	-

23	Brooks et al 2005	Bangladesh	2-12	809	812	35	7	52	Acetate	-	-

24	Heinig et al 2006	USA	4-10	41	44	5	7	28	Sulphate	-	-

25	Sazawal et al 2006	Tanzania	1-35	8120	8006	10	7	56	-	-	-

26	Long et al 2006	Mexico	6-15	389	198	20	7		Methionate	71.0	76.0

27	Richard et al 2006	Peru	0.5-15	428	215	20	7	28	Sulphate	-	-

28	Tielsch et al 2006	Nepal	1-36	7297	14241	10	7	68	Sulphate	-	-

29	Tielsch et al 2007	Nepal	1-35	20968	20308	10	7		Sulphate	-	-

30	Gupta et al 2007	India	6-48	854	858	25	2	26	-	-	-

31	Sazawal et al 2007	Tanzania	1-36	21274	21272	10	7	68	-	78.0	79.0

32	Bhandari et al 2007	India	1-23	47110	47249	10	7	52	Sulphate	64.0	64.2

33	Luabeya et al 2007	South Africa	4-6	202	105	10	7	72	Gluconate	77.2	78.6

34	Brown et al 2007	Peru	6-8	203	99	3	7	26	Sulphate	77.1	78.6

35	Fischer Walker et al 2007	India, Pakistan, Ethiopia	1-5	554	556	10	7	2	Sulphate	-	-

36	Chhagan et al 2009	South Africa	6-24	202	104	10	7	72	Sulphate	-	-

37	Taneja et al 2009	India	0	1026	1026	5	7	52	Sulphate	63.4	64.7


†Zn, zinc supplemented group; No Zn, zinc withheld group, details of the actual comparisons are provided in Supplementary Table (see Additional File 4)

* Mentioned as acetate/gluconate; -, not mentioned

### Analytical approach

We first attempted to classify the studies based on the outcomes they reported (Figure 1, right panel). We focused on five important and commonly reported outcomes: incidence of diarrhea, prevalence of diarrhea, incidence of persistent diarrhea, incidence of dysentery and incidence of mortality. For each outcome, we studied the reported effect sizes and the heterogeneity across studies. For these meta-analyses, we used the random effects model of DerSimonian and Laird [48].

For quantifying heterogeneity, we used two statistics: the I^2 ^statistic and the τ^2 ^statistic that represents the among-study variance. We also constructed 95% confidence intervals for the τ^2 ^statistic using non-parametric bootstrapping procedure [49] based on 5000 replicates. The two heterogeneity quantifying statistics have distinct merits in quantifying heterogeneity. The I^2 ^statistic can be compared across meta-analyses [50] and is related to the related τ^2 ^statistic such that it represents the estimated proportion of the total or marginal variance of a single study that is due to the among-study variance[51]. Also, if a trial only reported the Cochrane Q test result for heterogeneity then the I^2 ^statistic can be estimated from it using the formula I^2 ^= (Q-df)/Q with the minimum bound set to zero. On the other hand, there are two advantages of using the τ^2 ^statistic: first it can be used to estimate the 95% prediction intervals for the global distribution of the estimated summary effect measure. Second, it can be used to estimate the proportion of populations that are likely to show a relative risk exceeding unity[52,53]. This parameter is referred to here as the opposite affects proportion [denoted in the rest of the paper by Pr(OE)]. The theoretical details underlying these two uses of the τ^2 ^statistic are described in details in a supplementary note (see Additional File 3).

Publication bias was examined using funnel plot [54] and the regression intercept method described by Egger at al[55]. We also used Duval and Tweedie's trim and fill approach to examine the publication bias[56]. To examine if the existing evidence points towards a changing benefit of zinc supplementation, we conducted cumulative meta-analyses in which each subsequently published study was added to meta-analysis and the procedure of DerSimonian and Laird [48] method repeated iteratively. We evaluated all the included studies to identify possible risks of bias using the risk of bias assessment tool recommended by t the Cochrane Collaboration for Systematic Reviews [57]. The tool uses six questions (see legend to Figure six A) to which the answers can be summarized as no risk of bias, definite risk of bias or uncertain risk of bias. To quantify the overall risk of bias in a study we coded these three categories of responses as 1, 1.5 and 2, respectively and then summated the scores for all the six questions. Thus the total risk score varied in the range from 6 to 12 with a score of six indicating no risk of bias and a score of 12 indicating a highly biased study. We then assessed if this risk of bias score partly explained the variability in RR estimates across studies.

We investigated the potential contributors to the heterogeneity with a three pronged approach: First, for continuous variables (age, plasma zinc concentration and dose of zinc) we conducted univariate meta-regression analyses as recommended by Higgins et al [50] and Thomson et al [58] and attempted to quantify the extent of contribution of the predictors to the heterogeneity. Second, we conducted sub-group meta-analyses analyses for the following categorical variables: continent of origin, zinc salt, countries classified by income categories, zinc only studies, studies not included in previous meta-analyses and studies with age-range up to 12 months. For classifying the trial country into income groups, we used the country-specific estimates of the Gross National Income provided by the World Bank using the Atlas method. (http://data.worldbank.org/data-catalog) Third, the age of study subjects posed a special challenge in our meta-analysis. Most of the studies reported the age as a range rather than mean. For the meta-regression analyses explained in the first approach, we therefore used mid-point of the age range as an approximation for the average age in a study and used these mid-points for meta-regression. However, we also conducted a complementary analysis in which we conducted a set of restricted meta-analyses for each month of age. For example, if we wanted to estimate the benefit of zinc supplementation at 15 months of age then we included only those studies in which the age range straddled 15 months. We ran these restricted meta-analyses for each month of age over the range of 0-48 months and examined if the relative risk for the incidence of diarrhea was stable at all ages.

Another challenge to our analytical approach was the fact that some of the trials used a cluster-randomization procedure. It has been extensively described that the weights from a random effects model need to be appropriately adjusted to accommodate and overcome the potential influence of design effect on summary estimates in a meta-analysis[59,60]. We therefore, also adjusted the random-effects models for the design effect (which was reported in relevant trials) from cluster-randomized trials. The adjustment for design effect was done by multiplying the inverse-variance weight for a study by design effect. The design-effect for trials that did not use cluster randomization was treated as one. Statistical analyses were conducted using the Stata 10.2 (Stata Corp, College Station, TX) software package. For meta-analyses we used the metan.ado program written by Bradburn [61] whereas for the cumulative and restricted meta-analyses we used the metan.ado program iteratively through dedicated Stata scripts.

## Results

We included 37 identified studies (220,805 subjects) for reports on one or more of the following five outcomes: incidence of diarrhea, prevalence of diarrhea, incidence of persistent diarrhea, incidence of dysentery and incidence of mortality(Figure 1B). The number of studies included for meta-analysis were: 31 for incidence of diarrhea [7-12,17-21,23,25-28,30,31,33-39,41,43-47], 15 for prevalence of diarrhea [12,23,24,29,31-36,38,39,41,43,44], 11 for incidence of persistent diarrhea [21,30,33-36,38-40,45,46], 7 for incidence of dysentery [18,30,34,36,40,45,46] and 12 for incidence of mortality [10,11,18,23,29,32,42,43,45,46]. These studies represented 38, 16, 12, 8 and 14 distinct comparisons, respectively (Figure 1B). The details of the specified comparison groups for individual trials and the sources of these studies are given in the Supplementary Table 1 (see Additional file 4).

### Association of zinc supplementation with diarrheal incidence

**Figure 2:**
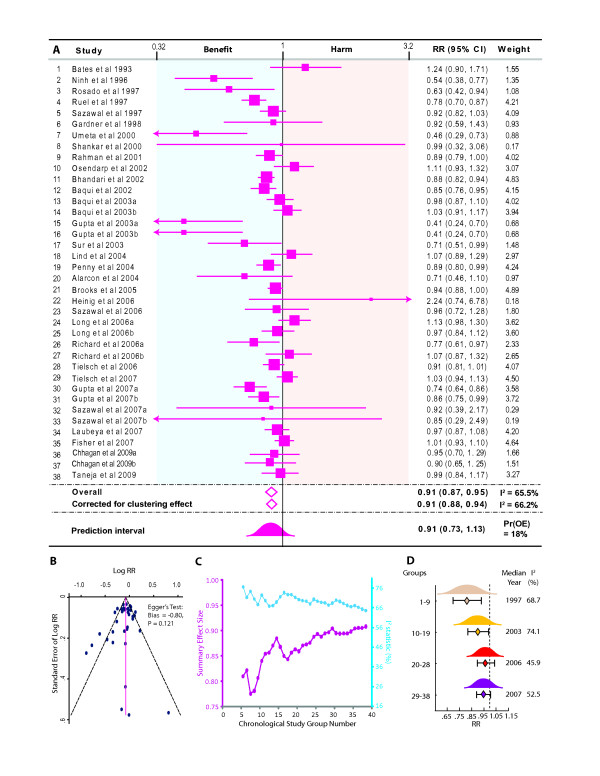
**Meta-analysis of the influence of preventive zinc supplementation on the incidence of diarrhea**. **(A) **Forest plot showing the point (squares proportional to study weight) and 95% confidence interval (error bars) estimates for each study. Colored background indicates harm (pink) or benefit (blue) of zinc supplementation. Summary relative risk estimate is shown as a diamond both by the random effects model ("Overall") and corrected for the design effect from cluster-randomized trial ("Corrected for clustering effect"). The 95% prediction interval (PI) is shown as a standard normal curve. Suffixes a and b indicate comparisons within a single study against the same placebo as detailed in Supplementary Table (see Additional File 4). RR, relative risk; weight, percentage weight; Pr(OE) opposite effects proportion. **(B) **Funnel plot for the investigation of publication bias. **(C) **Cumulative meta-analyses of chronologically ordered studies. Results are shown as the point estimate of the effect size (purple diamonds aligned to the left y-axis) and the I^2 ^statistic (blue squares aligned to the right y-axis). Results are shown from study group five onwards for which significant effects were observed **(D) **Contribution of calendar year to zinc supplementation. Results are represented as point estimates (diamonds) 95% CI (error bars) and 95% PI (standardized normal curves). Heterogeneity is shown as I^2^. The estimates for τ^2 ^were: study groups 1-9: 0.0232 (95% CI 0.0029-0.1407); study groups 10-19: 0.0143 (95% CI 0.0033-0.0712); study groups 20-28: 0.0055 (95% CI 0.0000-0.0313); and study groups 29-38: 0.0062 (95% CI 0.0000-0.0231).

The summary relative risk estimate for incidence of diarrhea was contributed by a total of 69934 and 75028 children in zinc and comparison groups, respectively. Since some studies used more than one groups compared to the same reference group, the total numbers of comparisons used in this meta-analysis were 38 as shown in Figure 2A. Our results indicated that there was a 9% [summary relative risk estimate size 0.91, 95% CI 0.87-0.95] lower incidence of diarrhea among children who received zinc supplementation (Figure 2A). The strength of this association did not change even after adjusting for the design effect from cluster-randomized trials (Figure 2A) and thus for the reason of simplicity we report the findings from a standard random effects model. The estimated value of τ^2 ^was 0.0107 (95% confidence interval 0.0049 - 0.0217). Using this value of the among-study variance indicated that the 95% prediction interval for the summary relative risk estimate was 0.73-1.13 and the Pr(OE) was 18% indicating that at the level of populations it may be premature to assume a clear benefit of zinc supplementation and that approximately 18% populations are likely to show a relative risk estimate exceeding unity.

We considered two potential sources for a potentially biased summary RR estimate in our meta-analysis. First, since zinc was supplemented during episodes of acute diarrhea in two trials [12,18] we considered whether inclusion of these trials could have influenced our meta-analysis. Sensitivity analyses (see Additional file 5) showed that exclusion of these two trials had negligible impact on the results of meta-analysis. Second, the funnel plot (Figure 2B) indicated that was no significant publication bias (Egger's test: bias = -0.8; p = 0.12; Figure 1B). We evaluated the publication bias using Duval and Tweedie's trim and fill approach also and found that no trimming was required and the results therefore corroborated those of the Egger's test.

To determine if there has been a temporal change in the observed benefit of zinc supplementation we conducted two sets of analyses. We first conducted cumulative meta-analyses for the summary relative risk estimate for incidence of diarrhea by iteratively including all studies that predated a specific trial. Significant benefit of zinc was observed onwards from the fifth study group. (Figure 2C) A striking observation from this cumulative meta-analysis was that there was trend for a monotonically decreasing benefit of zinc with the chronological rank of the studies. We thus considered the possibility of using calendar year of publication year as a predictor of the relative risk using meta-regression but could not conduct those analyses as the distribution of this variable was skewed towards more recent years (Shapiro-Wilk's p = 0.027). Alternatively, to keep the size of subgroups balanced, we categorized the trials into four classes based on the quartiles of the publication year. We observed (Figure 2D) that a significant protective benefit of zinc was observed in the first two quartiles of publication year but not in the third and fourth ones further substantiating the likelihood that the accumulation of more recent evidence points towards a diminished preventive benefit ascribable to zinc supplementation. To affirm this point further, we considered whether the trials included in our review that were not available for previous meta-analyses demonstrated a lack of beneficial association of zinc supplementation with diarrheal incidence. We observed that seven comparison groups from five new trials [7,8,11,12,62] indeed provided no evidence in favor of zinc supplementation [summary RR estimate 0.99, 95% CI 0.93 - 1.05] while the meta-analysis of the remaining 31 comparisons yielded statistically significant beneficial association with zinc supplementation [summary RR 0.90, 95% CI 0.85 - 0.95].

An alternative way to measure the potential influence of zinc on diarrheal incidence is to consider the outcome of multiple episodes as has been done for other conditions[51,63]. We thus examined if zinc supplementation affords a protection against occurrence of multiple episodes of diarrhea. Only four trials [12,26,33,64] explicitly stated this outcome. The remaining trials did not clearly state whether the incidence is reported for only first episodes or for subsequent episodes as well. Three trials [12,26,33] reported the outcome as occurrence of ≥2 episodes of diarrhea while the remaining trial [64] categorized the outcome as 0 episodes, 1-3 episodes, 4-6 episodes and >6 episodes. All the four trials suggested a strong protective association of zinc supplementation with multiple episodes of diarrhea. Meta-analytic summary of three trials [12,26,33] indicated that zinc supplementation afforded a 31% protection against occurrence of ≥2 episodes [summary relative risk estimate = 0.69, 95% CI 0.53 - 0.95, 95% prediction interval 0.44 - 1.09, Pr(OE) 1.9%] although there was heterogeneity in the results (I^2 ^68%, τ^2 ^0.0317, 95% CI for τ^2 ^0.0231 - 0.284).

### Investigation of heterogeneity across studies on diarrheal incidence

In both the overall and the cumulative meta-analyses there was a statistically significant heterogeneity of effects across the published studies (I^2 ^65.5%, τ^2 ^0.0107, 95% CI for τ^2 ^0.0049 - 0.0217). We therefore proceeded to investigate if the reported trial characteristics can partially explain this heterogeneity. Specifically, we examined the contribution of the following variables to heterogeneity: age, continent of origin, country economy (as a surrogate for prevalence of zinc deficiency), zinc salt, duration of zinc supplementation, dose of zinc supplementation and co-interventions. As shown in Table 1, only 12 of the included studies reported the baseline plasma zinc level and hence we could not the influence of this variable on heterogeneity.

**Figure 3:**
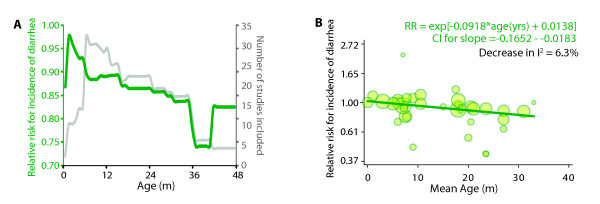
**Association of age of the study subjects with the benefit of zinc supplementation**. **(A) **Results from restricted meta-analysis for each month of age (x-axis). Restricted meta-analysis was conducted for each month of age by including only those studies in which the range of age of the study subjects straddled the selected month of age. Left y-axis (green) shows the relative risk for incidence of diarrhea while the right y-axis (grey) shows the number of studies included in meta-analysis **(B) **Meta-regression. In this analysis the mid-point of the age range for each study was meta-regressed onto the relative risk of observed diarrheal incidence in that study. Each circle represents a trial - the diameter of a circle is proportional to the standard error in that study. Dark green line is the predicted regression line. Meta-regression on this variable indicated that accounting for age would reduce the I^2 ^statistic by 6.3%. P_slope _indicates the significance value for the test of a two-tailed null hypothesis that the slope of the regression line is zero.

We examined the contribution of age to heterogeneity in three ways. First, the restricted meta-analysis for each month of age showed (Figure 3A) that the relative risk of diarrhea was strongly inversely correlated with age. We found that there was a strong positive correlation (Pearson's r = 0.82, p < 0.0001) between age and the summary relative risk for incidence of diarrhea indicating that the beneficial association of zinc supplementation, on an average, increased by 0.31% for each month of age starting from a benefit of 7.4% (95% CI 5.7% - 9.2%) at age zero. Maximum benefit of zinc supplementation (26%) was observed above 36 months but this association was contributed to by only 6 studies (Figure 3A). Second, using the alternative approach of meta-regression we found that age explained 6.3% of the heterogeneity across studies and had a significant association (p = 0.014) with the reported incidence of diarrhea (Figure 3B). Third, we categorized the included studies into two groups - those in which the age range was entirely below 1 year and the remaining studies. Subgroup meta-analysis using this dichotomization indicated (Table 2) that statistically significant association could not be found in studies recruiting all children less than one year of age. Thus, three complementary approaches implied that zinc supplementation may benefit more by restricting it to higher ages. It should be noted however, that the 95% prediction intervals were non-significant at all ages.

**Table 2:** Results of subgroup analysis for the outcome of diarrheal incidence

Characteristic	N	SRR	95% CI	95% PI	**I**^**2 **^**(%)**	**τ**^**2**^	**95% CI for τ**^**2**^	Pr(OE) %
Age category								

Age range < = 12 months	12	0.95	0.88-1.02	0.77-1.18	69.9	0.0099	0.0000 - 0.0345	30.3

Remaining studies	26	0.89	0.83-0.94	0.71-1.12	61.9	0.0114	0.0029 - 0.0284	13.8

Type of comparison*								

Zinc versus placebo	25	0.88	0.82-0.95	0.67-1.16	72.2	0.0173	0.0069 - 0.0404	16.6

Zinc+x versus x	5	0.90	0.85-0.96	0.84-0.96	6.7	0.0003	0.0000 - 0.0256	<0.001

Zinc+x+y versus y	8	0.98	0.82-1.04	0.80-1.20	21.2	0.0014	0.0000 - 0.0269	29.5

Continent of origin								

Africa	8	0.92	0.79-1.08	0.67-1.26	43.7	0.0185	0.0000 - 0.1702	27.0

Asia	19	0.91	0.86-0.96	0.74-1.12	72.2	0.0099	0.0038 - 0.0252	17.2

Americas	10	0.90	0.80-1.01	0.66-1.22	68.7	0.0192	0.0059 - 0.0756	22.4

Oceania	1	0.99	0.32-3.06	---	---	---	---	---

Country economy								

Low income	14	0.94	0.87-1.01	0.76-1.16	63.4	0.0089	0.0006 - 0.0393	25.6

Lower middle income	12	0.85	0.78-0.94	0.65-1.11	75.5	0.0154	0.0051 - 0.0540	9.5

Upper middle income	11	0.94	0.87-1.02	0.78-1.13	43.0	0.0068	0.0000 - 0.0294	22.7

High income	1	2.24	0.74-6.78	---	---	---	---	---

Zinc salt								

Acetate	5	0.96	0.89-1.04	0.83-1.11	54.3	0.0037	0.0000 - 0.0118	25.1

Gluconate	7	0.91	0.87-0.95	0.87-0.95	0.0	0.0000	0.0000 - 0.0026	---

Methionate	3	0.95	0.76-1.19	0.63-1.42	74.6	0.0267	0.0005 - 0.1466	37.8

Sulphate	18	0.84	0.85-0.93	0.60-1.18	75.2	0.0276	0.0100 - 0.0742	14.7

Not mentioned	5	0.88	0.78-1.00	0.69-1.13	60.0	0.0112	0.0000 - 0.1171	11.4

Duration of supplementation								

2-10 weeks	3	0.92	0.82-1.03	0.75-1.13	70.6	0.0067	0.0049-0.0124	15.4

10-26 weeks	15	0.85	0.78-0.93	0.63-1.14	75.5	0.0190	0.0056-0.0566	11.9

27+ weeks	20	0.94	0.89-1.00	0.78-1.13	51.2	0.0072	0.0000-0.0207	23.3

N, number of study groups; SRR, summary relative risk estimate; CI, 95% confidence interval based on DerSimonian and Laird model estimates; PI, 95% prediction interval [details in supplementary note (see Additional File 3)]; τ^2^, among-study variance; CI, confidence interval; Pr(OE), opposite effects proportion

*, For details please see text.

In the subgroup analyses, we found that the 19 study groups from Asia [11,12,18,19,21,23,25,26,28,33,34,41,44-46] showed a significant reduction in diarrheal incidence but with large heterogeneity among study groups. In contrast, studies from Americas [17,27,30,31,35,37-39], Africa [7-10,20,47] or Oceania [43] could not demonstrate a significant benefit of zinc for this outcome (Table 2). Corroborating these results, we also found (Table 2) that trials from the countries in the lower middle income group [11,12,25,26,28,39,43,44,64,65] demonstrated the strongest benefit of zinc supplementation while trials from countries in the upper middle [7,8,17,30,31,35,37,38] or high income group [27] did not demonstrate such a benefit. Surprisingly, we found that studies from the low income group countries [9,10,18-20,23,33,34,36,45-47] also failed to demonstrate beneficial utility of zinc against diarrheal incidence. With regard to zinc salt, majority of the studies used either zinc sulphate (18 study groups, [9,11,12,17,25,27,28,31,33,37,39,44-46]) or zinc gluconate (7 study groups, [7,8,21,35,43,65]). We found that the trials using zinc gluconate showed the most significant reduction in diarrheal incidence with no heterogeneity while the trials using zinc sulphate (and those which did not mention the salt used) also showed a statistically significant reduction in diarrheal incidence. The trials that used zinc acetate were homogenous but did not show a significant reduction in relative risk of diarrhea. Lastly, we found that the trials supplementing zinc for a duration of 10-26 weeks [17,19,21,25,26,28,31,33-35,41,47] showed a reduced likelihood of diarrhea but trials using zinc supplementation for shorter [12,18,36] or longer [7,8,10,11,20,23,27,30,37-39,43-46,62] duration than this interval did not show a statistically meaningful benefit for the outcome of diarrheal incidence.

**Figure 4:**
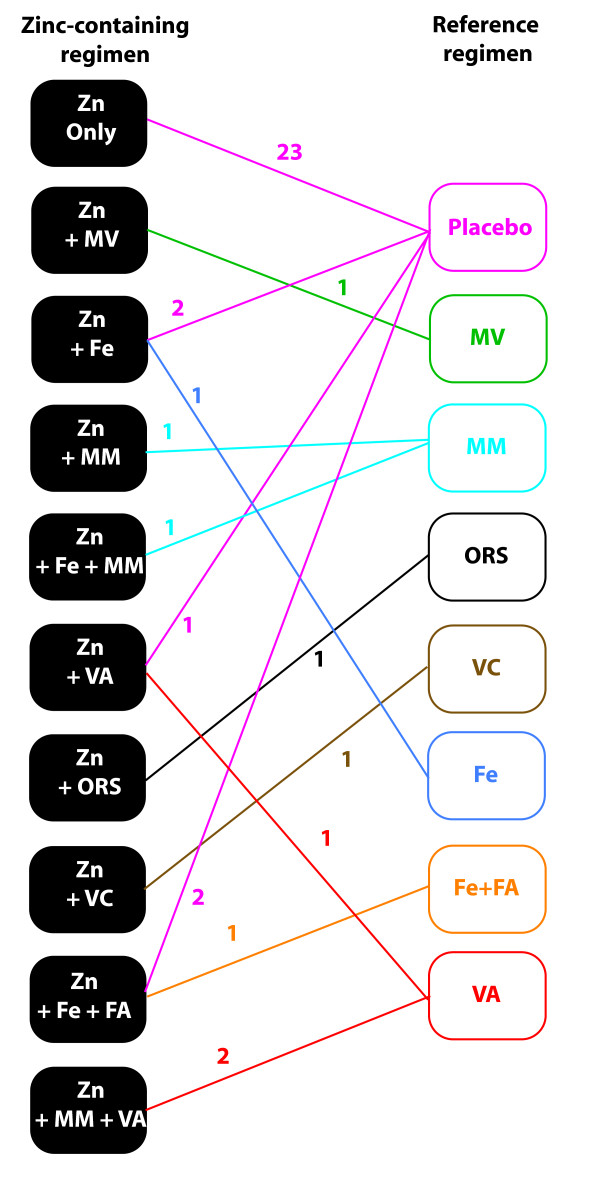
**Network diagram representing various comparisons found in the studies on diarrheal incidence**. Eight different reference groups are color coded and so are the comparisons emanating from these reference groups. Each line represents a comparison and the color-coded number alongside the line is the number of trials reporting the specified comparison.

One of the main challenges in our meta-analyses was the consideration of co-interventions. Figure 4 shows the network diagram for the various comparisons observed in the included studies. Formal network meta-analysis (mixed treatment comparisons) [66-70] could not be done because the treatment regimens used in various studies (Figure 4) cannot be considered fully discrete [69,70]. We thus classified the studies into three groups - those that compared zinc supplementation versus placebo,[7,9-12,20,23,25-28,30,31,33,34,36,37,39,43,44,47,64] those that compared the combination of zinc with some co-intervention that was also given to the reference group (referred to as zinc + x versus x in Table 2) [17-19,35,41] and those that combined two more co-interventions with zinc but gave only one co-intervention to the reference group (referred to as zinc + x + y versus y in Table 2) [7,8,19,30,37,38,45,46]. Interestingly, we observed that (Table 2) the benefit of zinc supplementation could be ascertained from the zinc versus placebo studies as well as the zinc + x versus x studies but in the remaining studies this association was not seen.

Next, using meta-regression we found that the dose of zinc (p = 0.321), the total amount of zinc received through supplementation (p = 0.467) or duration of zinc supplementation (p = 0.418) did not influence the observed benefit of supplementation and did not contribute significantly to the between-studies heterogeneity. This result from meta-regression also corroborated the results from subgroup analysis (Table 2) which showed that extremes of the duration of zinc supplementation were not associated with benefit of zinc supplementation. Together, our findings show that age, continent and zinc salt were the three important characteristics that could partly explain the significant heterogeneity of the beneficial association of zinc supplementation in diarrheal incidence.

**Figure 5:**
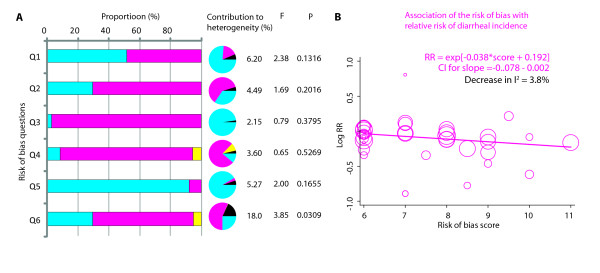
**Assessment and analysis of the risk of bias in studies**. **(A) **Six questions related to risk of bias were considered for each study (Q1-Q6). These were: Q1, Was the allocation sequence adequately generated? Q2, Was treatment allocation adequately concealed? Q3, Was blinding adequate? Q4, Was incomplete outcome data adequately addressed? Q5, Was there a selective outcome reporting? Q6, Is the study free of other biases? Answer to each question was either: no risk, high risk or uncertain. The bar chart shows, the proportion of each response for each question. Accompanying pie charts show the proportion of heterogeneity (overall Cochrane Q statistic) explained by each color coded response. The black slice in the pie shows the among-responses heterogeneity attributable to the bias being considered and is also given in percentage along side. F, Snedecor's F statistic; p, significance value for F. The F and p together indicate the statistical significance for the contribution of the bias to among-study heterogeneity. **(B) **Overall risk of bias as a contributor to among-study heterogeneity. The plot shows results of meta-regression of the overall risk of bias score with the log of relative risk. The risk of bias score ranged from 6 (no risk of bias) to 12 (high risk of bias). Each circle represents a trial - the diameter of a circle is proportional to the standard error in that study. Dark magenta line is the predicted regression line.

Lastly, we conducted risk of bias analyses to investigate if existing risks of bias in the included studies could provide clues into the source of among-study heterogeneity. Of the six potential biases investigated for, we observed that a compilation of "other biases" explained 18% of the among-study heterogeneity (p = 0.0309) while the remaining five biases did not individually afflict the among-study heterogeneity in a significant way. Details of these biases for each study are provided in Supplementary Table 2 (see Additional file 3). To consider whether these biases might influence the among-study heterogeneity in a concerted fashion, we meta-regressed the total risk of bias score on the log of reported RRs. We observed that (Figure 5B) there was trend for a more significant association of zinc supplementation with diarrheal incidence in studies that showed a higher risk of bias (p = 0.062). Interestingly, we also observed that there was a statistically significant correlation between mean age in the trial and the risk of bias (Pearson's r = 0.34, p = 0.038) and therefore when these two variables were together entered into a multivariate meta-regression model, both lost the statistical significance (data not shown). Together these analyses demonstrated that studies with high age group and (sometimes concomitant) higher risk of bias were likely to report a larger benefit attributable to zinc supplementation.

### Association of zinc supplementation with other outcomes

**Figure 6:**
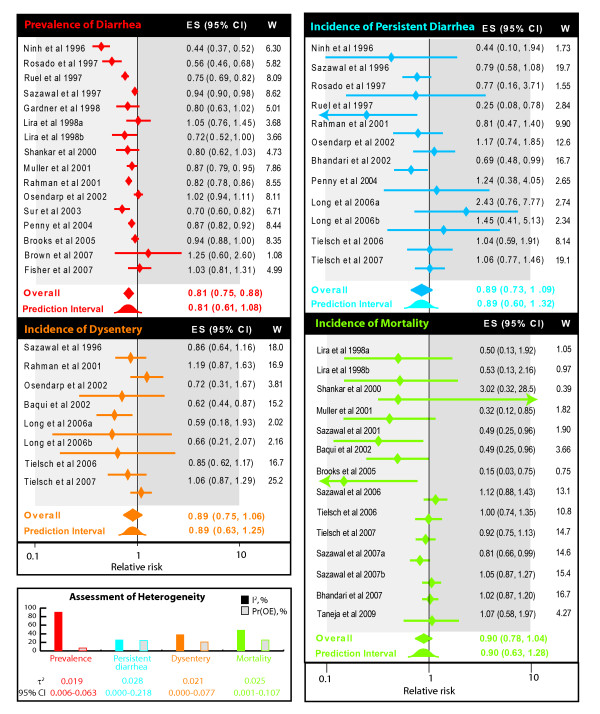
**Forest plots for the meta-analysis of the influence of preventive zinc supplementation on four diarrhea outcomes**- prevalence of diarrhea (red), incidence of persistent diarrhea (blue), incidence of dysentery (orange) and incidence of all-cause mortality (green). Diamonds and error bars indicate the point and 95% confidence interval observed in each study. ES, effect size; CI, confidence interval; W, statistical weight for derived from the DerSimonian and Laird random effects model. Summary relative risk estimate size for each outcome is shown as a filled diamond. The 95% prediction interval is shown as a standard normal curve and the opposite effects proportion [Pr(OE)] are shown in the box depicting assessment of heterogeneity. Suffixes a and b to some studies indicate two comparisons within a single study against the same placebo as detailed in Supplementary Table (see Additional File 4). Light grey background indicates beneficial effect of zinc and dark grey background indicates a harmful effect of zinc. Degree of heterogeneity across studies in the meta-analysis for each outcome is shown as color-coded pie-charts on the lower-left. The filled slice in the pie-charts indicates the I^2 ^statistic. Also shown are the values of τ^2 ^along with the 95% confidence interval, the among-study variance for each outcome.

We next evaluated the influence of zinc supplementation on the remaining four outcomes described in Figure 1. The summary relative risk estimate for prevalence of diarrhea was contributed by 15 studies [12,23,24,29,31-36,38,39,43,44,65] which enrolled 3501 and 3033 children respectively in zinc and comparison group without zinc. There was a 19% (summary relative risk estimate 0.81, 95% CI 0.75-0.88; Figure 6) lower prevalence of diarrhea among children who received zinc supplementation. However, there was significant heterogeneity of this effect as indicated by an I^2 ^statistic of 89.5%. For the outcome of incidence of persistent diarrhea 11 studies [21,30,33-36,38-40,45,46] with 12 distinct comparisons which enrolled 31106 and 36899 children in the zinc-supplemented and comparison group, respectively were included in our meta-analysis. Our results showed a non-significant association with on the reduction of risk by 11% (summary relative risk estimate 0.89, 95% CI 0.73-1.09) - a finding that was homogeneous across the included studies (I^2 ^25%, Figure 6). The summary relative risk estimate for reduction of incidence of dysentery was estimated from 7 studies [18,30,34,36,40,45,46] with 8 distinct comparisons of 33391 and 39446 children respectively in zinc and comparison groups. There was a non significant reduction of 11% (0.89, 95% CI 0.75-1.06, Figure 6) with an I^2 ^of 38% indicating homogeneity across studies. Finally, the effect of zinc on mortality was assessed from 12 studies [10,11,18,22,23,29,32,42,43,45,46,62] showing 14 distinct comparisons enrolling 111790 and 118140 children in zinc and comparison group. Although there was a 10% reduction in the risk of mortality (summary relative risk estimate 0.90, 95% CI 0.78-1.04, Figure 6) it did not achieve statistical significance and again the findings were homogeneous as indicated by an I^2 ^of 48% (Figure 4). For these four outcomes, we did not conduct subgroup meta-analyses or meta-regression owing to the small number of studies. For all these outcomes the 95% prediction intervals included a value of unity. For the outcome of prevalence of diarrhea, exclusion of the Fisher Walker et al trial [12] (due to zinc supplementation during acute diarrhea) did not influence the summary relative risk estimate while exclusion of the Baqui et al [18] trial for the same reason further reduced the likelihood of a beneficial association of zinc supplementation with dysentery. The details of these analyses are provided in supplementary meta-analyses (see Additional file 5).

## Discussion

We reviewed a total of 37 studies that reported the value of zinc supplementation in children for five different diarrhea-related outcomes. We first considered our findings in the light of the three previous meta-analyses of preventive trials [4-6] that had observed a 14-20% reduction in the incidence of diarrhea in zinc supplemented children. Since significant heterogeneity among trials was observed we compared the point estimates for effect sizes, their 95% confidence intervals, the total number of studies and children included in this study with those in the previously published meta-analyses (Table 3). Our estimate of the protective benefit of zinc supplementation was consistently lower that that reported in all previous meta-analyses. Further, the 95% prediction intervals of the summary relative risk estimate for all the five outcomes in this meta-analysis straddled unity. With respect to the outcome of diarrheal incidence however, it should be noted that this outcome essentially deals with all (not just the first) episodes of diarrhea during follow-up and that the three trials that explicitly studied the association of zinc supplementation with multiple episodes of diarrhea did demonstrate a significant benefit for the prevention of ≥2 episodes.

**Table 3:** Comparison of the results our study with those published previously

Outcome	Meta-analysis	RCTs	N	Statistic	Point Estimate	95% CI	**I**^**2 **^**(%)**
Incidence of diarrhea	M1	7	1502	OR	0.82	0.72 - 0.93	---

	M2	15	6272	RR	0.86	0.79 - 0.93	77.3

	M3	24	16665	RR	0.80	0.71 - 0.90	---

	This study	31	144962	RR	0.91	0.86 - 0.95	65.5

Prevalence of diarrhea	M1	7	1502	OR	0.75	0.63 - 0.88	---

	This study	15	6534	RR	0.81	0.75 - 0.88	89.5

Incidence of persistent diarrhea	M1	6	1770	OR	0.67	0.42 - 1.06	---

	M2	3	2585	RR	0.75	0.57 - 0.98	56.3

	This study	11	68005	RR	0.89	0.73 - 1.09	25.4

Incidence of dysentery	M1	3	884	OR	0.87	0.64 - 1.19	---

	M2	4	4250	RR	0.85	0.75 - 0.95	30.3

	This study	7	72837	RR	0.89	0.75 - 1.06	37.9

Mortality	M4	4	86152	RR	0.91	0.82 - 1.02	---

	M3	10	201616	RR	0.94	0.86 - 1.02	---

	This study	12	229930	RR	0.90	0.78 - 1.04	48.1

---, not reported

M1, Bhutta et al 1999[5]; M2, Aggarwal et al 2007[4]; M3, Brown et al 2009[6]; M4, Tielsch et al 2007[45]

OR, odds ratio; RR, relative risk; RCT, randomized controlled trial; CI, confidence interval

In this study, cumulative meta-analyses helped to sequentially update the summary relative risk estimate size by incorporating results from each newly available study and also assess its impact on the heterogeneity. We observed a steady decline in the protective afforded by zinc with a corresponding decline in the heterogeneity which however remained significant (I^2 ^= 65.5%) till the inclusion of the last study. Our investigation into the potential sources of the observed heterogeneity of results raises several interesting and important possibilities. First, since a distinct slope for the effect size is visualized (Figure 2C), it indicates instability of the pooled effect size[71,72]. This instability indicates that more and more negative studies (that is, studies reporting no clear benefit of zinc supplementation) have accumulated over the recent past (a finding affirmed by our subgroup analyses) and thus the clear benefit of zinc observed during early years appears to have shrunken or elapsed from more recent studies.

Second, it may be possible that there is a substantial variability in the microbiological spectrum of the causes of acute diarrhea across the studies. We have recently [73] reported that the therapeutic benefit of zinc is not equal against all organisms such that the *Klebsiella *spp were most responsive to zinc supplementation, *Esherichia coli *were neutral while rotavirus diarrhea was associated with worse outcomes in zinc supplemented children. It is conceivable that a similar phenomenon may be operative in the scenario of diarrhea prevention as well. This notion is also indirectly supported by our observations that a significant benefit of zinc is not universally observed across continents of study origin or countries classified on the basis of economy both of which may somewhat reflect the heterogeneity in the microbiological spectrum of causative organisms.

Third, in our meta-regression analyses, age was significantly associated with the magnitude of the beneficial association of zinc supplementation and explained 6% of the heterogeneity. The restricted meta-analyses showed a steep decline in the protection below 12 months of age (Figure 3A, B). The previous meta-analyses of 24 studies with 33 distinct comparisons also validated that the benefit of zinc supplements on diarrhea incidence was limited to studies of children with mean initial age greater than 12 months [6]. Considered in the light of the causal gamut of childhood diarrheas, it is likely that the more common pathogens at age below 12 months (for example, rotavirus infections) may be refractory to the benefit of zinc supplementation at least as observed in the context of diarrhea treatment. This finding indicates that zinc supplementation may benefit substantially by triaging on the basis of age.

Combined with these findings we also found that the studies that recruited higher age groups seemed also to be a higher risk of biases. Consequently, there is a putative circular relationship among age, bias and observed effect. Alternative interpretations are possible in such a scenario and the true interpretation is currently unknown. For example, on the one hand it can be argued (as stated thus far) that zinc may be beneficial at higher age in reducing the prevalence and incidence of diarrhea. On the other hand, it can also be argued that the observed benefit of zinc at higher ages may be influenced by implicit study biases and that more carefully done and reported studies in children with higher age are likely to shed more light on the magnitude of a true zinc benefit in diarrhea prevention.

Fourth, the sub-group analyses also showed that zinc salts may have a differential bearing on diarrheal incidence. Studies that used zinc gluconate showed homogeneity and significant reduction in diarrheal incidence (I^2 ^0%, summary relative risk estimate 0.90, 95% CI 0.86 - 0.94). Those using zinc sulfate were heterogeneous but showed a statistically significant reduction in diarrheal incidence (I^2 ^84.3%, summary relative risk estimate 0.75, 95% CI 0.63 - 0.89). There were fewer studies that used zinc acetate and showed no benefit of zinc but were homogenous. Elsewhere, we reported that a sub-group analyses of therapeutic effect of zinc in reduction of diarrheal duration also showed that studies using zinc gluconate had a significant homogeneous reduction in diarrheal duration although increased the risk of vomiting[74]. These findings beckon a reconsideration of the policy formulations regarding the salt to be used for large scale zinc supplementation program aimed at diarrhea prevention. Together, these findings from cumulative and sub-group analyses provide clues into the heterogeneity of results from various studies of zinc supplementation for diarrhea prevention.

It is noteworthy that Asia has the largest population of stunted children[75-77]. It is expected that those countries at high risk of zinc deficiency, i.e. prevalence stunting exceeding 20% and estimated prevalence of inadequate zinc intake of more than 25%, would most likely benefit from prophylactic and therapeutic zinc supplementation[78]. In this context it is interesting that our findings from the sub-group analyses that studies only from Asia demonstrated a favorable association of zinc supplementation albeit with significant heterogeneity. Despite the food insecurity and poverty in developing countries of both Asia and Africa, studies from Africa did not show a significant beneficial association of zinc in reducing the risk of diarrhea and were homogenous. Anthropometric indices, baseline plasma zinc levels or use of co-interventions may have differed between studies in these continents.

Our estimate of the influence of zinc on the prevalence of diarrhea was slightly lower than that reported by Bhutta et al[5] over a decade ago (Table 1). However, that outcome had the highest heterogeneity across studies and therefore, although appealing, the summary relative risk estimate size should be (metaphorically) taken with a pinch of salt. More homogeneous studies are needed before a conclusive impact of zinc can be deduced on prevalence of diarrhea. In contrast to the benefit of zinc for incidence and prevalence of diarrhea, we could not demonstrate a clear benefit of this intervention in reducing the incidence of persistent diarrhea, dysentery and mortality. Two previous meta-analyses that reported the effect of zinc on persistent diarrhea reached opposite conclusions (Table 3) - one [4] showed a significant effect while the other did not [5]. In contrast, therapeutic zinc supplementation during acute diarrhea has been reported to have a clear benefit in reducing the incidence of persistent diarrhea by ~25% and in reducing the proportion of children with persistent diarrhea > 3 days by ~30%[74]. This advantage of zinc supplementation however does not appear to be extending to prevention of persistent diarrhea. Similar inconsistency was also observed for the effect of zinc on the incidence of dysentery (Table 1). These results imply that prevention of persistent diarrhea and dysentery may not serve as realistic goals for preventive zinc supplementation.

Finally, Tielsch et al [45] conducted a meta-analysis of the effects of zinc supplementation on young child mortality using two reports from South Asia and two from Sub-Saharan Africa. The pooled estimated relative risk of mortality across all ages for zinc supplementation was 0.91 (95% CI 0.82-1.02). A post hoc analysis showed that zinc had a protective effect against mortality only in children over 12 months [OR 0.82, 95% confidence interval (CI) 0.70 - 0.96]; and in infants there was no effect (OR 1.04 95% CI 0.90 - 1.21). The mortality diminishing effects of zinc supplementation were also equivocal in the subsequent review which included 10 studies (Table 1)[6]. Our systematic review shows that adequately powered trials failed to demonstrate a significant mortality reducing association of zinc supplementation in children less than five years. Based on these data, we surmise that currently it is too premature to definitively conclude about the role of preventive zinc supplementation on mortality. However, it should also be borne that our meta-analysis as well as the two previous meta-analyses [6,45] have attempted to study the effect of zinc supplementation on all-cause mortality. It is possible that zinc supplementation may have more pronounced effect on diarrhea-related or pneumonia-related mortality [4,64] which may have been masked when considering all-cause mortality.

Our meta-analyses had several strengths. First, as shown in Table 3 our meta-analyses studied all the five outcomes which, to our knowledge, no other study has done in the past. Second, our meta-analyses were based on several additional studies that the previous reviews did not include. Third, cumulative meta-analyses included in our study provided additional insights into the chronological change in the pattern of zinc benefit. Finally we attempted to probe the observed heterogeneity and conducted meta-regression and sub-analyses to explain it. Our study also has several limitations. First, only a few studies reported information on most of the predictor variables simultaneously so multivariate meta-regression analyses could not be conducted to understand the importance of the factors identified in a multivariate context. Second, only covariates reported in the trials can be examined but conceivably other known and unknown predictors of zinc benefit may also partly explain the observed heterogeneity. Third, meta-regression and subgroup analyses, in themselves, cannot be considered as diagnostic tools but just provide clues into the possible sources of heterogeneity. Fourth, due to lack of reported data we could not fully tease out the potential influence of socio-economic and nutritional determinants of the differential benefit of zinc supplementation. Fifth, we consistently observed a high degree of heterogeneity across studies (sometimes even within subgroups). Such high degree of heterogeneity can be seen as a reason enough to question the validity of the summary effect measures which might reflect an over-simplification of the truth. Together, our findings urge that more focused investigation of the potential sources of heterogeneity is needed to refine and improve the benefits from zinc supplementation for prevention of diarrhea.

## Conclusions

We observed that zinc supplementation reduces incidence of diarrhea by 9% with more pronounced association observed beyond 12 months of age. We attempted to translate this benefit into a perceptible public health measure. A limitation of relative risk as a measure of association is that decision making also needs information on the extent of baseline risk. From a practical perspective, measures such as number needed to treat (NNT), which rely on the absolute rather than relative risk reduction, are more informative [79-82]. Using the results of our meta-analyses, we therefore estimated the number needed to prevent (NNP) diarrheal episodes. An NNP of unity indicates that all eligible children be provided the preventive intervention[83]. Using the mathematical relationship among relative risk, baseline risk, absolute risk reduction and NNP, we determined that an NNP of unity can be achieved if the prevalence of children with at least one diarrheal episode in year is 13.74% (95% CI 9.40% - 26.60%). Thus zinc supplementation of eligible children would be appropriate for this range of prevalence of diarrheal episodes. It should also be noted that we observed a significant benefit of zinc supplementation against multiple episodes of diarrhea. However our cumulative meta-analyses showed that the strength of benefit of zinc supplementation appears to be shrinking as more studies are getting published. Also, almost all the 95% prediction intervals reported in this meta-analyses seemed to straddle unity suggesting that a reproducible clear benefit of zinc supplementation cannot be confidently inferred from the existing evidence. Therefore the strategy of prophylactic zinc supplementation for diarrheal prevention needs to be fine-tuned. We also observed that zinc supplementation had no demonstrable benefit against incidence of persistent diarrhea, dysentery and mortality in children however it should also be noted that the summary relative risk estimate was consistently less than unity for all outcomes. There is a continued need to understand sources of heterogeneity between studies so that zinc supplementation targets the population that is most likely to benefit.

## Abbreviations

CI: Confidence interval; ES: Effect size; NNP: Number needed to prevent; NNT: Number needed to treat; PI: Prediction interval; PRISMA: Preferred Reporting Items for Systematic Reviews and Meta-Analyses; RCT: Randomized controlled trial; SRR: Summary relative risk; UNICEF: United Nations Children's Fund; WHO: World Health Organization;

## Competing interests

The authors declare that they have no competing interests.

## Authors' contributions

ABP conceptualized the study, carried our data abstraction and wrote the manuscript; MM conceptualized the study, carried our data abstraction, conducted some analyses and wrote the manuscript; NB conducted statistical analyses and helped during the initial writing of the manuscript; HK conceptualized the study, carried out data abstractions, conducted analyses and wrote the manuscript and all authors have read and approve the manuscript.

## Pre-publication history

The pre-publication history for this paper can be accessed here:

http://www.biomedcentral.com/1471-2334/11/122/prepub

## Supplementary Material

Additional file 1 **PRISMA 2009 checklist**. Checklist including relevant page numbers for identifying various components of the review Click here for file

Additional file 2 **PRISMA 2009 Flow Diagram**. Flowchart detailing the trial recruitment protocol used in this review Click here for file

Additional file 3 **Supplementary Note**. Supplementary Note describing the merits of the τ^2 ^statistic over the I^2 ^statistic for measurement of heterogeneity across studies Click here for file

Additional file 4 **Supplementary Tables**. Supplementary Table describing additional trial characteristics Click here for file

Additional file 5 **Additional Meta-analyses**. Additional meta-analyses by excluding two trials that used zinc supplementation during episodes of acute diarrhea Click here for file
