# Supplementary material for: What zinc supplementation does and does not achieve in diarrhea prevention: a systematic review and meta-analysis
Source: BMC Infect Dis. 2011 May 12;11:122. doi: 10.1186/1471-2334-11-122 (PMC3115868; doi:10.1186/1471-2334-11-122)
Supplement: Additional file 2 — PRISMA 2009 Flow Diagram. Flowchart detailing the trial recruitment protocol used in this review [file 1471-2334-11-122-S2.DOC]

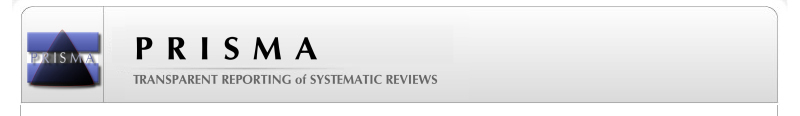
**PRISMA 2009 Flow Diagram For Selecting Published RCTs for the present Meta-Analysis**


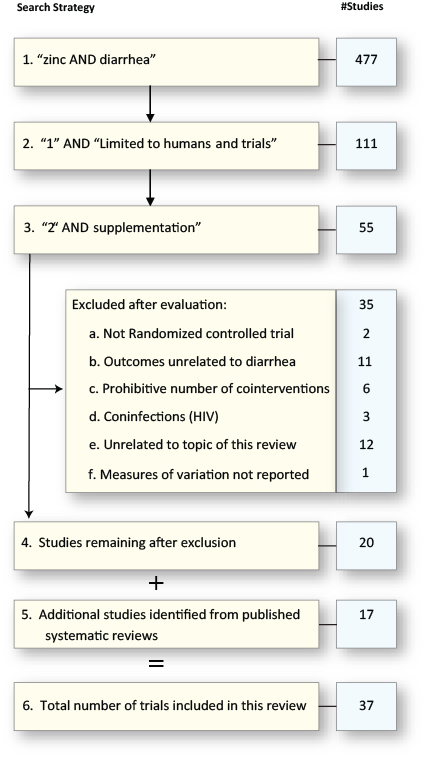


**Identification**

**Screening**

**Eligibility**

**Included**
