# Supplementary material for: What zinc supplementation does and does not achieve in diarrhea prevention: a systematic review and meta-analysis
Source: BMC Infect Dis. 2011 May 12;11:122. doi: 10.1186/1471-2334-11-122 (PMC3115868; doi:10.1186/1471-2334-11-122)
Supplement: Additional file 3 — Supplementary Note. Supplementary Note describing the merits of the τ2 statistic over the I2 statistic for measurement of heterogeneity across studies [file 1471-2334-11-122-S3.DOCX]

**Supplementary Note**

**Merits of the τ^2^ statistic over the I^2^ statistic**

In the random effects model of meta-analysis, it is assumed that the summary effect measure follows a normal distribution with the parameters µ and τ^2^ where µ represents the population mean and τ^2^ represents the among-study variance. For an intervention that is intended to be associated with a
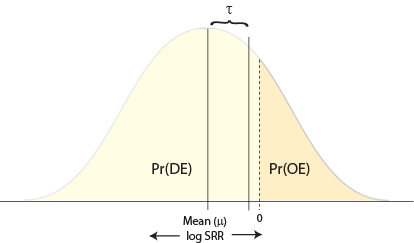
reduced risk of the disease outcome, such a distribution can be conceptually depicted as shown in the adjoining figure. Here, the bell-shaped symmetric curve represents the normal distribution of the relative risk estimates (RR) from different population that are clustered around the mean µ with a standard deviation of τ.[1] Considering the properties of a standard normal distribution, the information contained in τ can be represented in two different ways which overcome some limitations of the I^2^ statistic.

First it is possible to construct a 95% confidence interval (CI) for the population distribution of the summary effect size measure that is based on the estimates of µ and τ^2^ as follows: $Lower bound of 95\% CI=\mu-1.96\surd\tau^{2}$ and $Upper bound of 95\% CI=\mu+1.96\surd\tau^{2}$. This estimate of the confidence interval is different from the one returned by the DerSimonian and Laird method [2] and tends to be wider. A practical problem in determining this interval, however, is the fact, that the inferences are sample-derived and true population effects remain unknown. To tide over this, Higgins et al [1] have suggested the use of prediction intervals. The 95% prediction interval is given by $M^{*}\pm t_{df}^{\alpha}\sqrt{T^{2}+V_{M}}$, where M^*^ represents the estimated effect size, $t_{df}^{\alpha}$ represents the Student's t statistic for a given type I error rate (α) and degrees of freedom (df); T^2^ represents the sample estimate of τ^2^ and V_M_ represents the variance of the estimated effect size.

Second, theoretically a beneficial intervention should have a RR less than one - the yellow colored area in the Figure shown above which corresponds to the desired effect (DE) of the intervention. The complement of this likelihood (orange shaded area in the figure) is the probability that the estimated RR shows an opposite effect (OE) to that desired. The smaller the orange shaded area the more useful an intervention can be at the level of populations. The formula for estimating the opposite effects proportion is $\Pr\left( OE \right)=1-Ф(\frac{-\mu}{\sqrt{\tau^{2}}})$, where $Ф$ represents the standardized normal cumulative distribution function.[3]

***References***

1. Higgins JP, Thompson SG, Spiegelhalter DJ: **A re-evaluation of random-effects meta-analysis.** *J R Stat Soc Ser A Stat Soc* 2009, **172:**137-159.

2. DerSimonian R, Laird N: **Meta-analysis in clinical trials.** *Control Clin Trials* 1986, **7:**177-188.

3. Poole C: **Review of submitted manuscript.**; 2010.
