# Supplementary material for: What zinc supplementation does and does not achieve in diarrhea prevention: a systematic review and meta-analysis
Source: BMC Infect Dis. 2011 May 12;11:122. doi: 10.1186/1471-2334-11-122 (PMC3115868; doi:10.1186/1471-2334-11-122)
Supplement: Additional file 4 — Supplementary Tables. Supplementary Table describing additional trial characteristics [file 1471-2334-11-122-S4.DOCX]

**Supplementary Table 1**. Additional characteristics of the studies included in the meta-analyses.

| **#** | **Study** | **Comparisons** | **Source** | | | |
| --- | --- | --- | --- | --- | --- | --- |
|  |  |  | M1 | M2 | M3 | M4 |
| 1 | Bates et al 1993 | Zn svs placebo |  | X |  |  |
| 2 | Ninh et al 1996 | Zn vs placebo | X | X | X |  |
| 3 | Sazawal et al 1996^*^ | Zn+multivitamins vs multivitamins |  |  |  |  |
| 4 | Rosado et al 1997 | Zn or Zn+Iron vs placebo | X | X | X |  |
| 5 | Ruel et al 1997 | Zn vs placebo | X | X | X |  |
| 6 | Sazawal et al 1997 | Zn+multivitamins vs multivitamins | X | X | X |  |
| 7 | Gardner et al 1998 | Zn vs placebo | X |  | X |  |
| 8 | Lira et al 1998 | Zn vs placebo |  |  | X |  |
| 9 | Umeta et al 2000 | Zn vs placebo |  | X | X |  |
| 10 | Shankar et al 2000 | Zn vs placebo | X^a^ |  | X |  |
| 11 | Muller et al 2001 | Zn vs placebo |  | X | X | X |
| 12 | Sazawal et al 2001 | Zn and micronutrients OR Zn+Iron and micronutrients vs placebo |  |  | X |  |
| 13 | Rahman et al 2001^*^ | Zn OR Zn+ Vit A and placebo |  |  |  |  |
| 14 | Osendarp et al 2002 | Zn vs placebo |  | X | X |  |
| 15 | Bhandari et al 2002 | Zn vs placebo |  | X | X |  |
| 16 | Baqui et al 2002^*^ | Zn+ORS vs ORS |  |  |  |  |
| 17 | Baqui et al 2003 | a. Zn vs placebo |  | X | X |  |
|  |  | b. Zn+Iron vs placebo |  | X | X |  |
| 18 | Gupta et al 2003 | a. Zn 10mg daily vs placebo |  | X | X |  |
|  |  | b. Zn 50mg weekly vs placebo |  | X | X |  |
| 19 | Sur et al 2003 | Zn vs placebo |  | X | X |  |
| 20 | Lind et al 2004 | Zn or Zn+Iron vs placebo |  | X |  |  |
| 21 | Penny et al 2004 | Zn OR Zn+micronutrients vs placebo | X^b^ | X | X |  |
| 22 | Alarcon et al 2004 | ZN+Iron OR Zn+Iron+Vit A vs Iron only |  |  | X |  |
| 23 | Brooks et al 2005 | Zn vs placebo |  | X | X | X |
| 24 | Heinig et al 2006 | Zn vs placebo |  |  | X |  |
| 25 | Sazawal et al 2006 | Zn+Iron+FA verus placebo |  |  | X |  |
| 26 | Long et al 2006 | a. Zn vs placebo |  |  | X |  |
|  |  | b. Zn+Vit A vs placebo |  |  | X |  |
| 27 | Richard et al 2006^*^ | a. Zn vs placebo |  |  |  |  |
|  |  | b. Zn+Iron vs placebo |  |  |  |  |
| 28 | Tielsch et al 2006 | Zn OR Zn+Iron+FA vs placebo |  |  | X |  |
| 29 | Tielsch et al 2007 | Zn OR Zn+Iron+FA vs placebo |  |  | X | X |
| 30 | Gupta et al 2007 | a. Zn vs placebo (supplmentation period) |  |  | X |  |
|  |  | a. Zn vs placebo (post-supplmentation period) |  |  | X |  |
| 31 | Sazawal et al 2007 | Zv vs placebo |  |  | X | X |
| 32 | Bhandari et al 2007 | Zn+Iron+FA vs FA |  |  | X |  |
| 33 | Luabeya et al 2007^*^ | Zn+Vit A OR Zn+Vit A+micronutrients vs Vit A |  |  |  |  |
| 34 | Brown et al 2007 | Zn+MV+ceral porridge vs cereal porridge |  |  | X |  |
| 35 | Fischer Walker et al 2007^*^ | Zn vs placebo |  |  |  |  |
| 36 | Chhagan et al 2009^*^ | a. Zn+Vit A vs Vit A |  |  |  |  |
|  |  | b. Zn+Vit A+micronutrients vs Vit A |  |  |  |  |
| 37 | Taneja et al 2009^*^ | Zn+Vit A vs Vit A |  |  |  |  |

M1, Bhutta et al 1999[1]; M2, Aggarwal et al 2007[2]; M3, Brown et al 2009[3]; M4, Tielsch et al 2007[4]

OR, odds ratio; RR, relative risk; RCT, randomized controlled trial; CI, confidence interval

^*^These studies have not been included in any meta-analysis published thus far

^a^This study was published after the M1 meta-analysis which included the data on the basis of an abstract.

^b^Preliminary data from this study was published in 1999 and was included in M1.

**References for published meta-analyses**

1. Bhutta ZA, Black RE, Brown KH, Gardner JM, Gore S, Hidayat A, Khatun F, Martorell R, Ninh NX, Penny ME, et al: **Prevention of diarrhea and pneumonia by zinc supplementation in children in developing countries: pooled analysis of randomized controlled trials. Zinc Investigators' Collaborative Group.** *J Pediatr* 1999, **135:**689-697.

2. Aggarwal R, Sentz J, Miller MA: **Role of zinc administration in prevention of childhood diarrhea and respiratory illnesses: a meta-analysis.** *Pediatrics* 2007, **119:**1120-1130.

3. Brown KH, Peerson JM, Baker SK, Hess SY: **Preventive zinc supplementation among infants, preschoolers, and older prepubertal children.** *Food Nutr Bull* 2009, **30:**S12-40.

4. Tielsch JM, Khatry SK, Stoltzfus RJ, Katz J, LeClerq SC, Adhikari R, Mullany LC, Black R, Shresta S: **Effect of daily zinc supplementation on child mortality in southern Nepal: a community-based, cluster randomised, placebo-controlled trial.** *Lancet* 2007, **370:**1230-1239.

**Supplementary Table 2**. Risk of bias evaluation in the studies included in the meta-analyses.

|  |  | **1** | **2** | **3** | **4** | **5** | **6** |
| --- | --- | --- | --- | --- | --- | --- | --- |
| **Sr. No.** | **Study** | **Allocation sequence adequately generated** | **Allocation adequately concealed** | **Blinding** | **Addressing of incomplete outcome data adequately** | **Selective outcome reporting** | **Free of Other bias** |
| **1** | **Bates et al 1993** | No | No | Yes | Uncertain | No | No |
| **2** | **Ninh et al 1996** | No | No | Yes | Yes | Yes | No, Not free of other bias as it is conducted only stunted children, 32% drop out rate, per protocol analysis and monthly mothers recall |
| **3** | **Sazawal et al 1996** | Yes | Yes | Yes | Yes | No | No, as other bias present as per protocol analysis and no adj. for confounding variables. |
| **4** | **Rosado et al 1997** | No | No | Yes | Yes | No | No, biases present as diarrhea defined by mother and not adjusted for confounding variables |
| **5** | **Ruel et al 1997** | No | No | Yes | Yes | No | Uncertain |
| **6** | **Sazawal et al 1997** | No | No | Yes | Yes | No | No, as Bias may be there due to pre protocol analysis. 30% attrition |
| **7** | **Gardner et al 1998** | No | No | Yes | Yes | Yes | No, not free of biases, as can be present as conducted in malnourished children, small sample and no adjustment for confounding |
| **8** | **Lira et al 1998** | No | No | Yes | No | No | No, not bias free as groups not balanced at baseline and 50% of children who received zinc were non randomly allocated |
| **9** | **Umeta et al 2000** | No | No | Yes | Uncertain | No | Yes free from other bias except for per protocol analysis |
| **10** | **Shankar et al 2000** | Yes | Yes | Yes | Yes | No | Yes biases taken care of but for the per protocol analysis |
| **11** | **Muller et al 2001** | Yes | Yes | Yes | Yes | No | Yes, but diarrhea definition is uncertain. |
| **12** | **Sazawal et al 2001** | yes | yes | Yes | Yes | No | Yes free of bias |
| **13** | **Rahman et al 2001** | No | Yes | Yes | Yes | No | No not bias free as information on adj. for confounders in analysis not given and per protocol analysis |
| **14** | **Osendarp et al 2002** | No | Yes | Yes | Yes | No | Yes bias free |
| **15** | **Bhandari et al 2002** | Yes | Yes | Yes | Yes | No | Yes bias free |
| **16** | **Baqui et al 2002** | No | No | No | No | No | Not bias free. Not a double blind placebo controlled trial, no allocation concealment, per protocol analysis, outcome based on unblinded mother's recall. |
| **17** | **Baqui et al 2003** | No | No | Yes | Yes | No | Yes bias free |
| **18** | **Gupta et al 2003** | No | Yes | Yes | Yes | No | Yes bias free |
| **19** | **Sur et al 2003** | No | Yes | Yes | Yes | No | May not be bias free as not controlled for confounding variables. |
| **20** | **Lind et al 2004** | Yes | Yes | Yes | Yes | No | Yes bias free |
| **21** | **Penny et al 2004** | No | No | Yes | Yes | No | Yes bias free |
| **22** | **Alarcon et al 2004** | Yes | Yes | Yes | Yes | No | Yes bias free |
| **23** | **Brooks et al 2005** | Yes | Yes | Yes | No | No | Not bias free due to differential withdrawal and numbers analyzed unclear. Baseline adj of variables also unclear. Repeated episodes treated independently using Poisson regression. |
| **24** | **Heinig et al 2006** | No | Yes | Yes | yes | No | Yes bias free other than adj in analysis. |
| **25** | **Sazawal et al 2006** | Yes | Yes | Yes | Yes | No | Yes bias free |
| **26** | **Long et al 2006** | No | Yes | Yes | Yes | No | Yes bias free |
| **27** | **Richard et al 2006** | Yes | Yes | Yes | Yes | No | Yes bias free |
| **28** | **Tielsch et al 2006** | Yes | Yes | Yes | Yes | No | Yes bias free |
| **29** | **Tielsch et al 2007** | Yes | Yes | Yes | Yes | No | Yes bias free |
| **30** | **Gupta et al 2007** | No | Yes | Yes | No | No | No, not free as not adj for confounders and not mentioned how many dropped out |
| **31** | **Sazawal et al 2007** | Yes | Yes | Yes | Yes | No | Yes bias free |
| **32** | **Bhandari et al 2007** | No | Yes | Yes | Yes | No | Yes bias free |
| **33** | **Luabeya et al 2007** | Yes | Yes | Yes | Yes | No | Not bias free as not adj for confounders |
| **34** | **Brown et al 2007** | Yes | Yes | Yes | Yes | No | Yes bias free |
| **35** | **Fischer Walker et al 2007** | Yes | Yes | Yes | Yes | No | Yes bias free |
| **36** | **Chhagan et al 2009** | Yes | Yes | Yes | Yes | No | Yes bias free |
| **37** | **Taneja et al 2009** | Yes | Yes | Yes | Yes | No | Yes free of other bias |
