# Supplementary material for: What zinc supplementation does and does not achieve in diarrhea prevention: a systematic review and meta-analysis
Source: BMC Infect Dis. 2011 May 12;11:122. doi: 10.1186/1471-2334-11-122 (PMC3115868; doi:10.1186/1471-2334-11-122)
Supplement: Additional file 5 — Additional Meta-analyses. Additional meta-analyses by excluding two trials that used zinc supplementation during episodes of acute diarrhea [file 1471-2334-11-122-S5.DOCX]

**Meta-analyses by excluding trials that supplemented zinc during acute diarrhea**

Of the 37 trials that were included in this study, two trials [1, 2] had supplemented zinc during the treatment of acute diarrhea. Conceptually, these studies can therefore contribute to summary relative risks that can be biased. To investigate this possibility, we conducted meta-analyses by excluding these two trials. At least one of these two trials reported the following three outcomes: incidence, prevalence and incidence of dystentery. The results for these three outcomes are shown here:

***Outcome: Diarrheal Incidence***

Following figure shows the forest plot for this outcome.


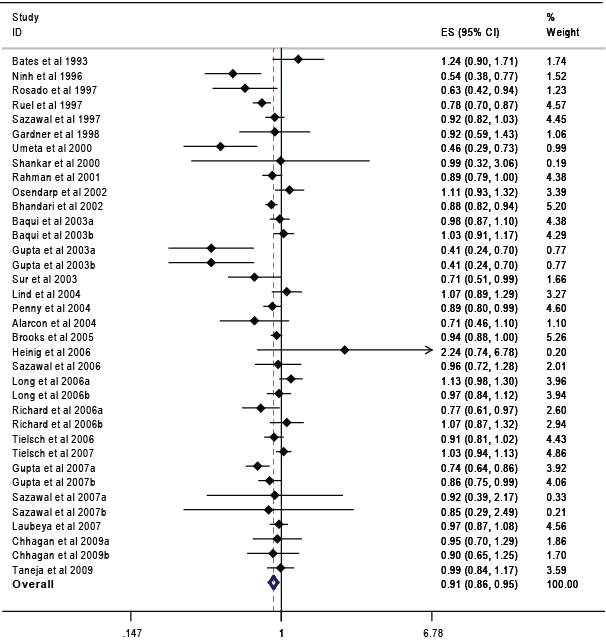


The I^2^ was = 65.3%, τ^2^ was 0.0115 (95% confidence interval 0.0049-0.0242) and the 95% prediction interval was 0.73-1.14. These results are negligibly different from those shown in Figure 2A.

***Outcome: Prevalence of diarrhea***

This figure shows the forest plot after excluding one trial [2] (the other trial [1] did not report prevalence).


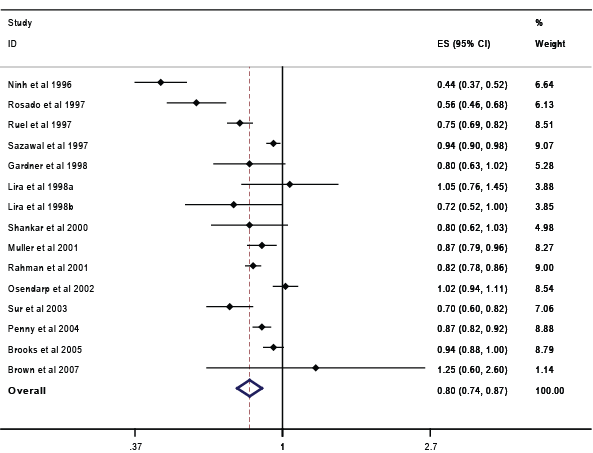


For this analysis, the I^2^ was = 90.0%, τ^2^ was 0.0195 (95% confidence interval 0.0059-0.0645) and the 95% prediction interval was 0.60-1.07. These results are highly concordant with those shown in Figure 5.

***Outcome: Dysentery***

The following figure represents the forest plot for this dataset after excluding one trial [1]:


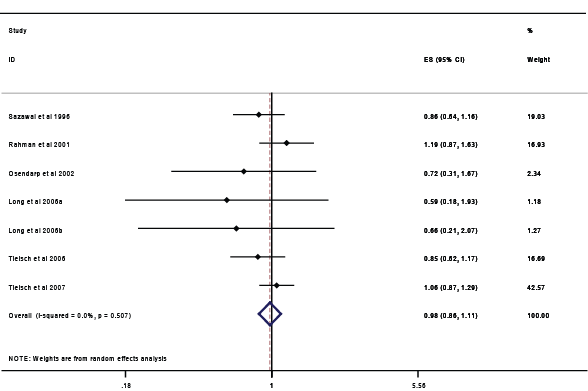


For this dataset, the I^2^ was and τ^2^ were 0 and thus the 95% prediction interval matched the 95% confidence interval shown in the forest plot. These results when compared with those shown in Figure 5 indicate that exclusion of this single trial did influence the summary relative risk estimate (towards null). However, this exclusion did not alter any inference since no beneficial association of zinc supplementation in dysentery was inferred even when the trial was included in meta-analysis as shown in Figure 5.

**References**

1. Baqui AH, Black RE, El Arifeen S, Yunus M, Chakraborty J, Ahmed S, Vaughan JP: **Effect of zinc supplementation started during diarrhoea on morbidity and mortality in Bangladeshi children: community randomised trial.** *Bmj* 2002, **325:**1059.

2. Walker CL, Bhutta ZA, Bhandari N, Teka T, Shahid F, Taneja S, Black RE: **Zinc during and in convalescence from diarrhea has no demonstrable effect on subsequent morbidity and anthropometric status among infants <6 mo of age.** *Am J Clin Nutr* 2007, **85:**887-894.
